# Thigh abscess and necrotizing fasciitis following an inside-out transobturator tape intervention: a case report

**DOI:** 10.1186/s13256-016-0942-3

**Published:** 2016-06-02

**Authors:** Jad Kerbaj, Camille Aubry, Caroline Prost, Philippe Brouqui

**Affiliations:** 1 Maladies Infectieuses et Tropicales, CHU Nord, AP-HM, Pôle des Maladies Infectieuses et Tropicales, Marseille, France; 2 Aix Marseille Université, URMITE, UM63, CNRS 7278, IRD 198, Inserm 1095, 13005 Marseille, France; 3 Institut Hospitalo-Universitaire Méditerranée Infection, 13005 Marseille, France; 4 Service d’imagerie Médicale, CHU Nord, AP-HM, Marseille, France

**Keywords:** Abscess, Necrotizing fasciitis, Vaginal transobturator tapes, *Staphylococcus aureus*

## Abstract

**Background:**

Tension-free vaginal transobturator tapes are used worldwide in the treatment of urinary incontinence in women. Very few severe complications have been described following this procedure, with no standard treatment yet established.

**Case presentation:**

We present the case of a 36-year-old French white woman with no remarkable medical history, presenting with an abscess and necrotizing fasciitis 48 hours after an inside-out tension-free transobturator procedure. Samples were collected by guided puncture from the abscess, retrieving *Staphylococcus aureus* and *Citrobacter koseri.*

**Conclusions:**

Severe complications following this procedure are rare, although it can have the potential for significant morbidity and even mortality, which is worth highlighting. We recommend early surgical treatment in combination with broad-spectrum antibiotics and coverage for *Staphylococcus aureus,* which may be a causative agent.

**Electronic supplementary material:**

The online version of this article (doi:10.1186/s13256-016-0942-3) contains supplementary material, which is available to authorized users.

## Background

Stress urinary incontinence is a frequent disabling pathology in women. Several methods of surgical treatment have been described; the most commonly used are tension-free vaginal tapes (TVTs) and transobturator tapes. Among the latter, there exists in particular the inside-out tension-free vaginal transobturator tape (TVT-O) and outside-in transobturator tape (TOT) [1]. Multiple studies have shown that these procedures have equivalent efficacy over the short and middle term [2, 3]. Tape infections are a very rare complication of this type of surgery, and it has been reported with both techniques [4]. In the TVT-O technique, a small incision is made in the vagina just below the opening of the urethra and a permanent tape is introduced via the vagina and placed under the urethra. The trocars used to introduce the tape are removed through small incisions at both sides of the upper inner thigh.

Multiple studies have been conducted over the last decade to assess the efficacy and complications of this surgical treatment [4]. Tape infection following this procedure has been rarely described, usually several months after the intervention. Of 2543 surgeries with 11 different tape systems, the Austrian registry recorded seven abscesses, none of them with the TVT-O [5]. The Norwegian registry reported four cases of infection, including simple urinary tract infection, after a TVT-O intervention in a population of 731 patients, with no severe infections [6]. The French registry records no major infections in a study containing a population of 984 women treated with TVT-O [7].

Here we describe an abscess of the adductor muscle of the left thigh and necrotizing fasciitis appearing 2 days after a TVT-O procedure in a 36-year-old woman.

## Case presentation

A 36-year-old French white woman with no underlying chronic conditions and a medical history of spontaneous pneumothorax presented to our institution for a motor deficit of the proximal portion and pain in her left lower limb with fever, which appeared 48 hours after implantation of a TVT-O for stress urinary incontinence (we did not know if she received any perioperative antibiotics). She took an anti-inflammatory drug (ketoprofen 50 mg, three times a day) for 4 days prior to admission to our hospital.

**Fig. 1:**
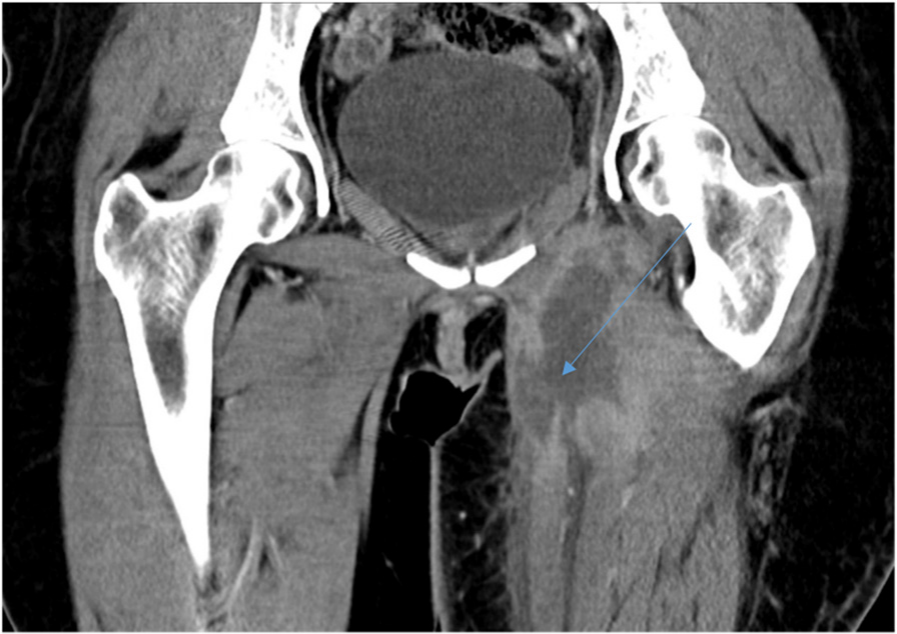
Abscess of the left adductor muscle (sagital section)

**Fig. 2:**
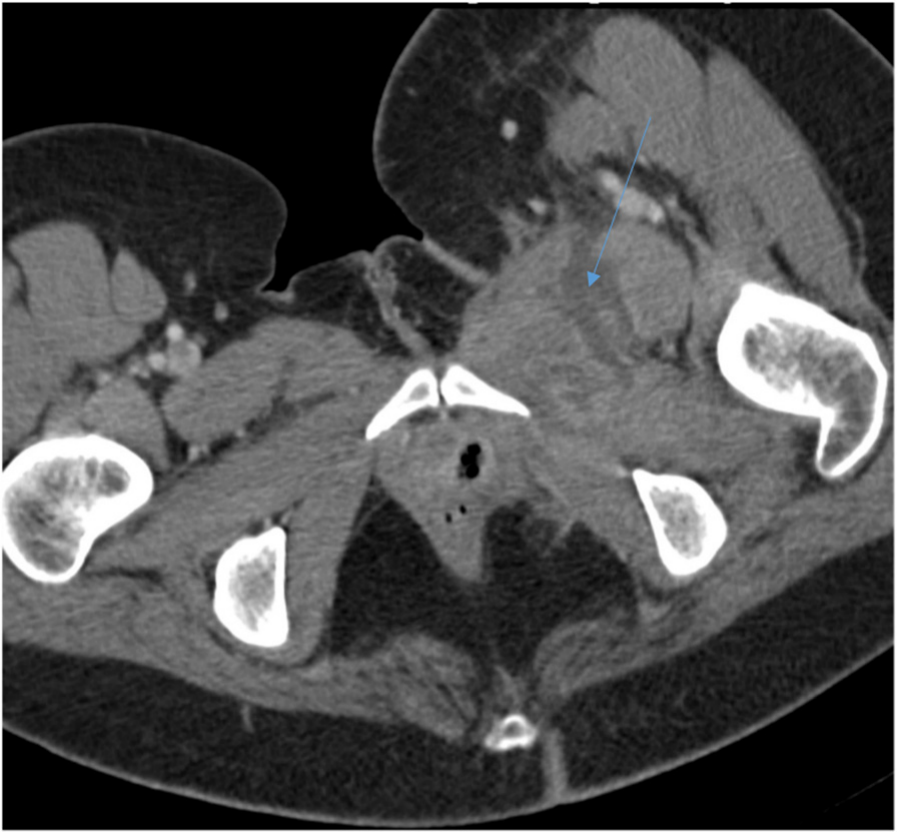
Abscess of the left adductor muscle (transverse section)

**Fig. 3:**
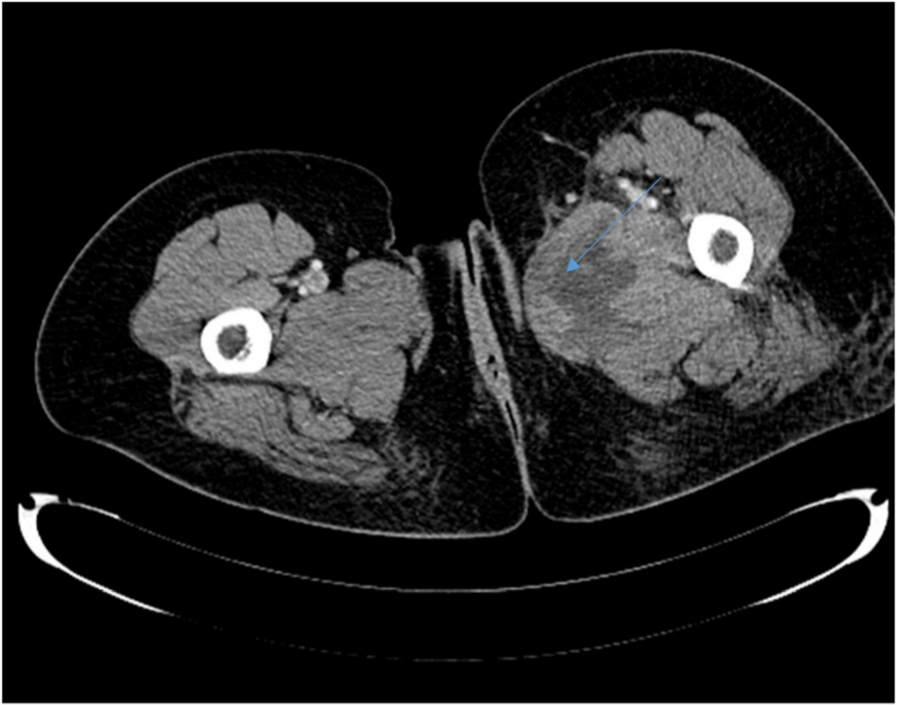
Left paraurethral abscess connecting with the left adductor muscle abscess

On clinical examination we did not find cellulitis or a collection, and she reported pain on palpation of her left thigh adductors. We performed a computed tomography (CT) scan on day 3 of her admission, which revealed an abscess of her left adductor muscle, measuring 53×28 mm (Figs. 1 and 2), and the presence of a fistula between the abscess and the transobturator tape (Fig. 3). Blood tests demonstrated very high inflammation parameters: C-reactive protein 300 mg/L and leukocytosis 18.54 giga/L [see Additional file 1: Table S1]. As she was clinically stable, we did not initiate antibiotic therapy before sampling the abscess.

On day 7, we sampled the abscess under ultrasound guidance. The liquid was purulent, and piperacillin-tazobactam 4 g three times a day and vancomycin 30 mg/kg/day intravenous treatment was initiated.

Two days later a purulent vaginal discharge appeared; our patient was still febrile. Surgery was indicated to allow local lavage and resection of the left part of the tape; the remaining transobturator tape could not be removed. The next day a necrotizing fasciitis of her inner and posterior left thigh appeared, her thigh was swollen, her skin was discolored and crepitus was present; she had tachycardia at 110 beats/minute and her blood pressure was 110/60 mmHg. A second laparotomy was conducted, with drainage and surgical lavage of the abscess. No hyperbaric oxygen was used due to her history of spontaneous pneumothorax.

The guided puncture cultures as well as the blood cultures were positive for methicillin-sensitive *Staphylococcus aureus. Citrobacter koseri,* sensitive to ampicillin-clavulanic acid, piperacillin-tazobactam, ceftriaxone, gentamycin, ciprofloxacin, and co-trimoxazole was recovered in the guided puncture culture. We adjusted the treatment to clindamycin 600 mg three times a day and piperacillin-tazobactam 4 g three times a day the same day. The local and general outcomes were favorable; we switched to oral antibiotic therapy with rifampicin 300 mg three times a day and ofloxacin 200 mg three times a day after 1 week and she was discharged. She had a monthly follow-up; the follow-up CT scan 1 month after discharge showed a complete disappearance of the abscess.

No vaginal discharge, pain, or fever was reported; the antibiotic therapy was given for a total duration of 2.5 months.

## Conclusions

Severe infections following a tension-free vaginal transobturator tape procedure are very rare; the fact that the incisions are small and that the surgery is of a very short duration greatly reduces the infectious risk [8]. These infections can carry potential for significant morbidity and even mortality, which is worth highlighting. Cases of abscesses, necrotizing fasciitis, and osteomyelitis have already been described [9–11].

Three case reports were found in the literature describing the presence of an abscess after the implantation of a TVT-O (research done on PubMed and Google Scholar): one obturator and thigh abscess occurring 2 years after a TVT-O procedure [9], one osteomyelitis 3 months after the implant [10], and one necrotizing fasciitis shortly after a TVT-O procedure [11]. However, multiple case reports relating TOT to severe infections were found in the literature; the microbiological findings were diverse, including: *Pseudomonas putida*, methicillin-resistant *S. aureus* [12], *Bacteroides fragilis* [13], and group B streptococcus [14]. Despite the fact that few cases and sparse microbiological documentation have been found for severe infections following TVT-O procedures, given the fact that the operation uses an endogenous pathway, causative agents are likely to be polymicrobial, and our case report indicates that they can also include *S. aureus*. In severe infections following transobturator slings we recommend early surgical drainage, with the removal of all foreign implants when possible, consideration of the use of hyperbaric oxygen treatment if there is no contraindication, as it is associated with a significant reduction in mortality in necrotizing soft tissue infections [15], and a broad-spectrum antibiotic covering *S. aureus* as a possible causative agent and subsequent adjustment of the empiric antibiotic therapy.

## Additional file

Additional file 1: Laboratory abnormalyties during patient care. (XLSX 9 kb)

## Abbreviations

CT: computed tomography
TOT: outside-in transobturator tape
TVT: tension-free vaginal tape
TVT-O: inside-out tension-free vaginal transobturator tape
